# Epigenetic Drift Is Involved in the Efficacy of HBV Vaccination

**DOI:** 10.3390/vaccines12121330

**Published:** 2024-11-27

**Authors:** Francesca Ferraresi, Simona Anticoli, Stefano Salvioli, Chiara Pirazzini, Luciano Calzari, Davide Gentilini, Christian Albano, Reparata Rosa Di Prinzio, Salvatore Zaffina, Rita Carsetti, Paolo Garagnani, Anna Ruggieri, Katarzyna Malgorzata Kwiatkowska

**Affiliations:** 1 Department of Chemistry “Giacomo Ciamician”, University of Bologna, 40126 Bologna, Italy; 2 Istituto Superiore di Sanità, Center for Gender Specific Medicine, 00161 Rome, Italy; simona.anticoli@iss.it (S.A.); anna.ruggieri@iss.it (A.R.); 3 Department of Medical and Surgical Sciences (DIMEC), University of Bologna, 40126 Bologna, Italy; stefano.salvioli@unibo.it (S.S.); chiara.pirazzini5@unibo.it (C.P.); 4 IRCCS Azienda Ospedaliero-Universitaria di Bologna, 40138 Bologna, Italy; 5 Bioinformatics and Statistical Genomics Unit, Istituto Auxologico Italiano IRCCS, 20095 Cusano Milanino, Italy; l.calzari@auxologico.it (L.C.);; 6 Department of Brain and Behavioral Sciences, University of Pavia, 27100 Pavia, Italy; 7 B Cell Unit, Immunology Research Area, Ospedale Pediatrico Bambino Gesù IRCCS, 00146 Rome, Italyrita.carsetti@opbg.net (R.C.); 8 Occupational Medicine/Health Technology Assessment and Safety Research Unit, Clinical-Technological Innovations Research Area, Ospedale Pediatrico Bambino Gesù IRCCS, 00146 Rome, Italysalvatore.zaffina@opbg.net (S.Z.)

**Keywords:** hepatitis B, vaccine, B cells, immune response, epigenetics, epivariants, epigenetic aging

## Abstract

**Background/Objectives**: HBV infections can lead to serious liver complications that can have fatal consequences. In 2022, around 1.1 million individuals died from HBV-related cirrhosis and hepatocellular carcinoma. Vaccines allow us to save more than 2.5 million lives each year; however, up to 10% of vaccinated individuals may not develop sufficient protective antibody levels. The aim of this study was to investigate the epigenetic drift in the response to HBV vaccine in isolated B cells. **Methods**: Epigenetic drift was measured by counting rare DNA methylation variants. These epivariants were detected in epigenome-wide data collected from isolated B cell samples from 41 responders and 30 non-responders (age range 22–62 years) to vaccination against HBV. **Results**: We found an accumulation of epivariants in the NR group, with a significant increase in hyper-methylated aberrations. We identified the chromosomes (1, 3, 11, 12, and 14) and genes (e.g., *RUSC1_AS1* or *TROVE2*) particularly enriched in epivariants in NRs. The literature search and pathway analysis indicate that such genes are involved in the correct functioning of the immune system. Moreover, we observed a correlation between epigenetic drift and DNA methylation entropy in the male population of the cohort. Finally, we confirmed the correlation between epivariant loads and age-related epigenetic clocks. **Conclusions**: Our findings support the idea that an age-related derangement of the epigenetic architecture is involved in unresponsiveness to the HBV vaccine. Furthermore, the overall results highlight the interconnection between various epigenetic dynamics (such as drift, clocks, and entropy), although these interconnections seem not to be involved in the altered immunological activity.

## 1. Introduction

Although most HBV infections are asymptomatic, acute or chronic infections may lead to liver impairment and consequently to hepatitis, cirrhosis, hepatocellular carcinoma, and eventually death. In 2022, the HBV death toll reached 1.1 million cases worldwide, mostly from cirrhosis and hepatocellular carcinoma (primary liver cancer) [1]. Since HBV is a blood-borne pathogen, healthcare workers are especially at risk for infection due to continuous occupational exposure to patients’ body fluids.

It is estimated that vaccines save more than 2.5 million lives each year, representing the most cost-effective health intervention. Immune protection generated by vaccination is the result of a complex interaction between innate, humoral, and cell-mediated immunity. There is a notable inter-individual (qualitative and quantitative) variation in the response to the HBV vaccine. Even though several factors such as sex, age, comorbidities, and sociocultural factors have been identified as shaping the immunological response [2], a deeper understanding of these influencing conditions and the mechanisms of their actions might bring opportunities to further optimize the immunogenicity and efficacy of vaccines and improve immunization strategies.

In particular, there is growing interest in studying the role of the epigenetic architecture and conservation of immune cells. A growing body of evidence shows that epigenetic aging influences immune response performance [3,4]. These results are coherent with a huge body of literature indicating that accelerated aging correlates with the derangement of physiological functions and disease development.

Recently, concepts such as epigenetic drift, epigenetic shift, antigenic drift, and antigenic shift became more and more frequent subjects of investigation. Even though there are some similarities, these are distinct phenomena (Supplementary Table S1). The epivariants calculated in the present study are classified as stochastic epigenetic mutations. Their burden, or cumulative presence, is recognized as an indicator of epigenetic drift because it increases continuously and exponentially over time. Due to their stochastic nature (occurring randomly without specific environmental or physiological triggers), these epivariants are not considered epigenetic shifts. Additionally, antigenic drift and antigenic shift were not examined in this study, as they pertain specifically to viral evolution and immune evasion rather than epigenetic changes in cells or organisms.

In the landscape of epigenetic studies, the measurement of epigenetic drift is of growing interest. Epigenetic drift is measured on DNA methylation data and is the count of the number of DNA methylation outliers per single individual [5,6]. These aberrant DNA methylation values have been defined as epivariants. There is increasing consensus that the accrual of such epivariation leads to the impairment of the correct epigenetic regulation of cell function, which, in sturn, contributes to eroding individual health. Interestingly, and coherently, it has been demonstrated that the epigenetic drift increases with age [5], mirroring, from the entropic perspective, the dynamic of biological clocks.

In the present study, we aimed to measure epigenetic drift, expressed with stochastic epigenetic mutations, or epivariants, in isolated B cells from individuals immunized with the HBV vaccine to investigate whether the distribution of epivariants is involved in the response to vaccination stimuli.

## 2. Materials and Methods

### 2.1. Participants and Phenotype

In this study, we analyzed a cohort of healthcare workers at the Bambino Gesù Children’s Hospital (Rome, Italy) who received vaccination against HBV, which is required by Italian law [7]. The studied cohort was described previously [3].

People who were born to a mother who was HBsAg+, had an HBV infection, or had not received anti-HBV vaccination were excluded from the study according to the experimental design.

All of the participants (*n* = 71) received an assessment of the protective immune response against the virus through anti-HBs titers. Individuals were classified as responders (Rs; *n* = 41; median age: 32 years; range between 22 and 53 years; females 35%) if their anti-HBs titers were at least 10 mIU/mL or higher.

If the anti-HBs titers were below 10 mIU/mL after the primary and the second vaccination cycle, they were identified as non-responders (NRs; *n* = 30; median age: 32 years; range between 22 and 62 years; females 50%). Antibody levels were measured 1 month after the last dose of the second cycle.

### 2.2. Sample Processing

From each participant, we collected PBMC samples and isolated B cells (RosetteSep Human B Cell Enrichment Cocktail; Stemcell Technologies, Vancouver, BC, Canada). We extracted (QIAamp DNA Blood Mini Kit; Qiagen, Hilden, Germany) and bisulfite-converted (EZ-96 DNA Methylation Kit Deep-Well; Zymo Research, Irvine, CA, USA) DNA to assess the genome-wide methylation (Infinium Human MethylationEPIC BeadChip; Illumina, San Diego, CA, USA).

### 2.3. Epivariant Calling and Outlier Detection

We performed quality control, probe cleaning (detection *p*-value < 0.01), and Noob normalization of the produced data (minfi v1.32.0; R v3.6.3). We converted normalized intensities into β-values and filtered out non-specific, cross-reactive, masked, variant containing, and X/Y chromosome-located probes [8,9,10].

The object of our analysis was stochastic epigenetic mutations, also known as epivariants [5]. Their burden, or cumulative presence, is a recognized indicator of epigenetic drift, since it increases continuously and exponentially over time. Due to their stochastic nature (occurring randomly without specific environmental or physiological triggers), these epivariants are different from, and are not considered, epigenetic shifts.

The epivariant calling was performed with an interquartile range (IQR) approach [5,6,11,12]. In brief, we identified the reference population-based range as [Q1 − 3 × IQR; Q3 + 3 × IQR], where IQR is the probe-wise interquartile range (Q3 − Q1) calculated using data from the R group, with Q1—25th percentile and Q3—75th percentile.

To differentiate between epivariants, hypo-epivariants, hyper-epivariants, and non-epivariants, we used this common IQR-based method involving the evaluation of the interquartile range of DNA methylation levels across specific genomic regions. In the studies by Gentilini et al. [5,6,11,12], the IQR was calculated to assess the methylation variability at specific loci, enabling the classification of regions based on their deviation from the median value. Therefore, following concepts were applied:

- Epivariants: These are characterized by methylation changes that significantly deviate from the population reference IQR, indicating substantial epigenetic divergence from normal levels.
- Hypo-epivariants: These show a reduction in methylation levels that fall below the lower quartile (or minimum IQR threshold), reflecting a loss of methylation compared to the baseline.
- Hyper-epivariants: These show an increase in methylation levels that fall below the upper quartile (or maximum IQR threshold), reflecting a gain of methylation compared to the baseline.
- Non-epivariants: These regions maintain methylation levels within the population reference IQR, indicating epigenetic stability without significant variation.

By using the IQR as a measure of dispersion, Gentilini et al. were able to identify epigenetic variations that deviate from the norm with greater precision, allowing for robust and statistically significant differentiation among epigenetic variant categories.

We applied the Hampel filter to detect outliers and remove samples with calculated epivariant counts outside of the interval [median − 3 × MAD; median + 3 × MAD], where MAD is the median absolute deviation.

### 2.4. Analysis

For each individual, the following three scores were calculated: (i) the total number of epivariant counts (TotEpiMut), (ii) the number of hypo-epivariant counts (HypoEpiMut), and (iii) the number of hyper-epivariant counts (HyperEpiMut). All of the scores were log-transformed a priori to ensure the normal distribution of the variable (Shapiro–Wilk test *p*-value < 0.05).

We investigated the relationship between epigenetic drift and unresponsiveness to the HBV vaccine by applying a generalized linear regression model (glm) and appropriately adjusting the data for age. This adjustment was crucial, as it allowed for the isolation of the effect of epigenetic drift independently from the simple passage of time. As a result, any association identified between epigenetic drift and vaccine response was corrected for age-related epigenetic changes, making the findings more reliable for specifically assessing the influence of epigenetic drift on immune response to the vaccine.

We examined the distribution of epivariants in relation to CpG islands and gene architecture. With an age-adjusted glm model, we evaluated the presence of significant differences in methylation aberration scores between the R and NR groups on global, chromosomal, and genetic levels. Sex, B cell count, and the array batch were included in the model as covariates. Nominal *p*-values were corrected for multiple tests with the Benjamini–Hochberg approach. By annotating all of the emerged probes, we generated separated lists of genes that carried epivariations in Rs and NRs. For genes that were exclusive for each phenotypic group, we identified significantly enriched pathways (Fisher’s exact test *p*-value < 0.05) using the Enrichr online tool [13] with the KEGG database [14] as a reference.

We extended the analysis by exploring if epivariant burden is related to other epigenetic aging phenomena associated not only with aging but also with several phenotypes and disorders. Therefore, for each sample, we estimated the global loss of homogeneity in DNA methylation by calculating Shannon’s entropy (agrmt v1.42.12; R v3.6.3), and we accessed five epigenetic clocks (DNAmAge, DNAmTL, DunedinPoAm, AltumAge, CRP_CpG_risk_score, PCHorvath1, and PCDNAmTL) that were previously predicted for the studied cohort [3]. We evaluated the correlations with a linear regression model (lm), correcting for phenotype, age, sex, B cell count, and the array batch.

Finally, considering the major differences between females and males observed in the immune response to viral vaccines [15], we also looked for the sex-specific differences between the R and NR groups. For this purpose, as previously, the glm model was applied, but separately in the population of females and males, and age, B cell count, and the array batch were used as the covariates.

## 3. Results

### 3.1. Characteristics of Cohort

The analysis of epivariants in the immunological response to the HBV vaccine was performed in 41 responders and 30 non-responders (Table 1). Phenotypic groups were sex- and age-matched, where 66% of the R subjects were females and were 33 years old on average, while 50% of the NR individuals were females and were 36 years old on average. Details on the cohort’s age distribution are provided on Supplementary Figure S1. There were no significant differences in the B cell counts in the samples collected from both groups.

### 3.2. Epivariant Distribution

In this further refinement of the previous analysis [3], we found, in total, 25,269 unique epivariants in R subjects and 19,357 unique epivariants in NR subjects (Supplementary Figure S2). A total of 23% of epivariants were located in the CpG island, around 20% were on the shores, 8% were mapped to the shelves, and 50% were mapped to regions outside of the islands (Supplementary Figure S3a). A total of 76% of the emerged probes were genic, of which 43% were located in the gene body (Supplementary Figure S3b). The distribution of the overall epivariants in relation to the CpG island and gene architecture was comparable between Rs and NRs (Supplementary Table S2).

The distribution patterns of the hypo- and hyper-methylated epivariants differed remarkably (Supplementary Figure S4). Notably, the great majority of hypo-methylated aberrations were accumulated in open-sea regions, while only a minority were located in the CpG island. Conversely, hyper-methylated epivariants tended to be located within and outside of the CpG island. In both cases, the remaining probes were distributed symmetrically between the shelves and shores. In relation to the genes, the rate of hypo-epivariants compared to hyper-epivariants was much higher in the gene body, while the hyper-methylated loci were more distributed in extremities zones (TSS1500, TSS200, 5′URT, and 1stExon). The observed patterns were maintained in both phenotypic groups (Supplementary Figures S5 and S6).

### 3.3. R and NR Differences in Epivariant Scores

#### 3.3.1. Global Level

**Figure 1:**
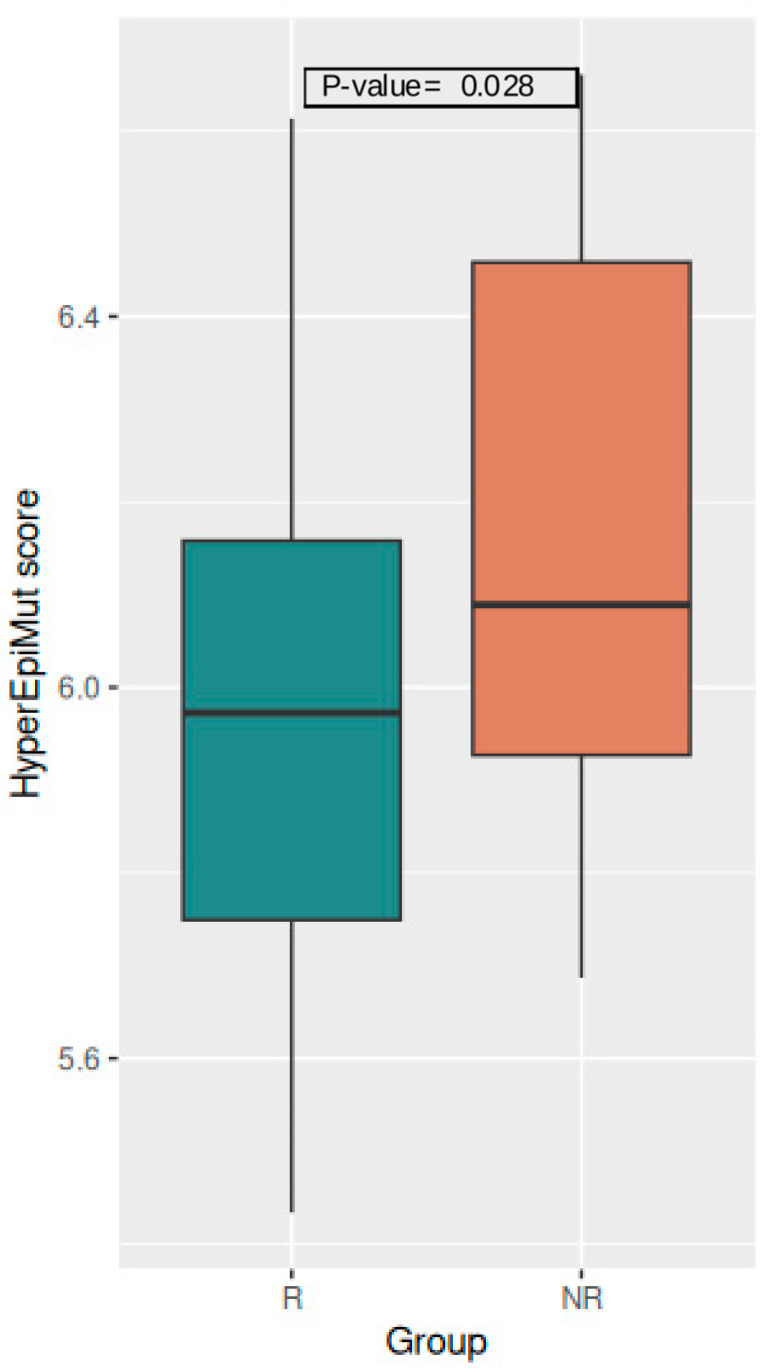
Boxplot of the HyperEpiMut score in the Rs and NRs.

The results of the epivariant score analysis on a global level revealed a tendency in the NR group to show higher values. The mean TotEpiMut score, comprising all epivariants, reached 6.78 in the R group and 6.88 in the NR group; however, this difference was not significant (*p*-value = 0.054; Supplementary Figure S7a). Considering the two types of epivariants separately, while the HypoEpiMut score was indifferent between the two groups (mean values R = 6.14 and NR = 6.14; *p*-value = 0.866; Supplementary Figure S7b), the values of the HyperEpiMut score resulted in being significantly higher among NRs compared to Rs (Rs = 5.98 and NRs = 6.17; *p*-value = 0.028) (Figure 1). Although the differences between the Rs and NRs in the sex-specific analysis did not reach the level of significance, the general trend was reproduced (Supplementary Figures S8 and S9).

#### 3.3.2. Chromosome Level

**Figure 2:**
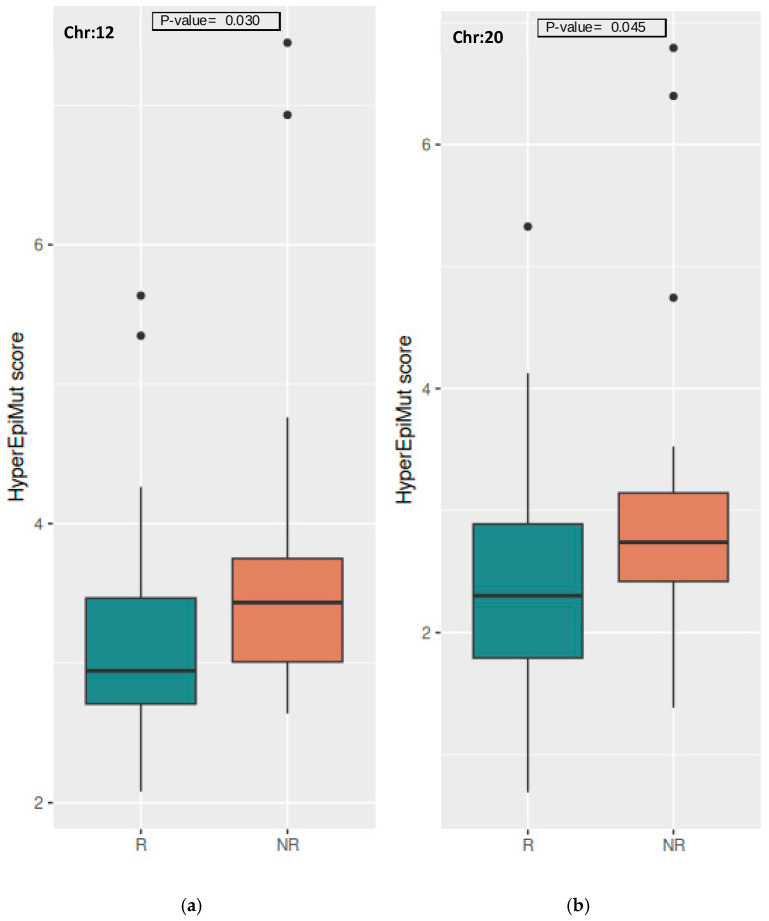
Boxplot of HyperEpiMut scores on the chromosomal level in the Rs and NRs for (**a**) chromosome 12 and (**b**) chromosome 20. Dots beyond the whiskers mark outliers with values beyond 1.5*IQR from either end of the Q1-Q3 box.

Our analysis also revealed the presence of differences between the Rs and NRs at chromosomal level. Compared to the Rs, the NRs showed higher TotEpiMut scores on chromosomes 12 (*p*-value = 0.017), 14 (*p*-value = 0.021), 1 (*p*-value = 0.031), 11 (*p*-value = 0.032), and 3 (*p*-value = 0.032) (Supplementary Figure S10a; Supplementary Table S3). The HyperEpiMut scores on chromosomes 12 (*p*-value = 0.030) and 20 (*p*-value = 0.045) were significantly higher in the NRs compared to the Rs (Figure 2; Supplementary Figure S10b; Supplementary Table S3). In females, the differences between the R and NR groups were accumulated on chromosomes 10 (*p*-value = 0.027), 11 (*p*-value = 0.034), and 21 (*p*-value = 0.041), and were related to the TotEpiMut scores (Supplementary Table S4). For males, the differentiating scores were the HyperEpiMut scores on chromosomes 17 (*p*-value = 0.036), 7 (*p*-value = 0.040), 12 (*p*-value = 0.041), and 14 (*p*-value = 0.049) (Supplementary Table S5). Regarding the hypo-epivariants, there were no differences between the two groups in the joined and sex-separated analyses. All of the reported *p*-values on the chromosomal level did not remain significant after multiple test corrections.

#### 3.3.3. Gene Level

We further explored the chromosomes for which significant differences between the Rs and NRs in epivariant scores were found, and we looked specifically for the differences on the gene level. Genes that emerged differentially enriched in the TotEpiMut and HyperEpiMut scores are listed in Table 2. Chromosomes that resulted in being significant in the sex-specific analysis allowed us to identify genes that were differentially enriched in NRs in either females or males (Table 3).

### 3.4. Pathways Associated to HBV Vaccine Response

When annotating the epivariants, we found 11,336 genes in the Rs and 9459 genes in the NRs. A total of 6578 of these epivariant-genes were common for both groups. Ranking the genes according to the total number of aberrant occurrences, the *CCM2L*, *SLC25A38*, *ARMC12*, *LOC285847*, and *NUMB* genes were the top five exclusively observed in the NRs, while the *UBE2T*, *LMF1-AS1*, *KIAA0430*, *CBR1*, and *LOC100133286* genes were the top five exclusively observed in the Rs. The pathway enrichment analysis of the list of NR-specific genes returned “Other glycan degradation”, “Pentose and glucuronate interconversions”, “Ascorbate and aldarate metabolism”, and “Steroid hormone biosynthesis” (Table 4). “Peroxisome”, “TNF signaling”, and “FoxO signaling” pathways were found among the KEGG terms enriched with R-exclusive epivariant genes (Table 5).

### 3.5. Epivariants and Epigenetic Aging

**Figure 3:**
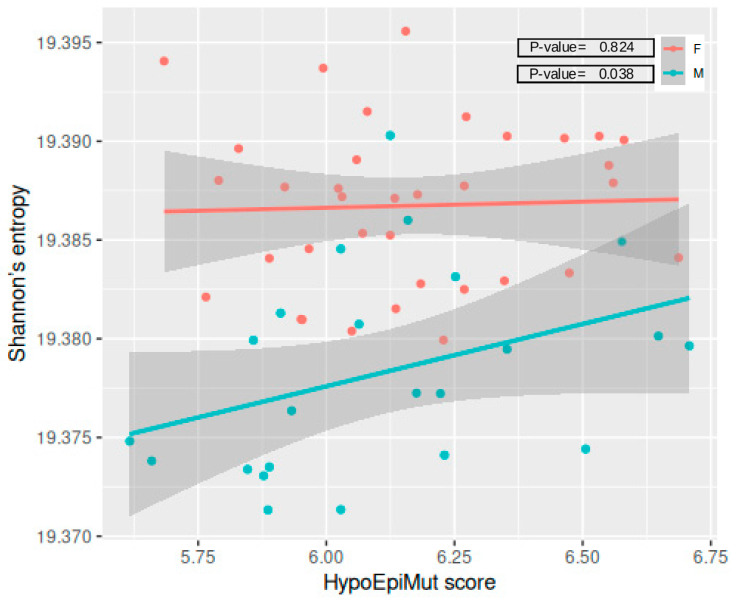
Correlation between Shannon’s entropy and the HypoEpiMut score in females and males. Each dot corresponds to a single sample, continuous lines represent linear regression trends for each sex group and the shadows outline respective standard error boundaries.

Shannon’s entropy did not differ between the Rs and NRs in our cohort, neither in the joined nor in the sex-specific analyses (Supplementary Figure S11); however, it resulted in being positively correlated with the HypoEpiMut score in the male population (*p*-value = 0.038) (Figure 3). There were no other significant correlations found with the TotEpiMut and HyperEpiMut scores (Supplementary Table S6).

**Figure 4:**
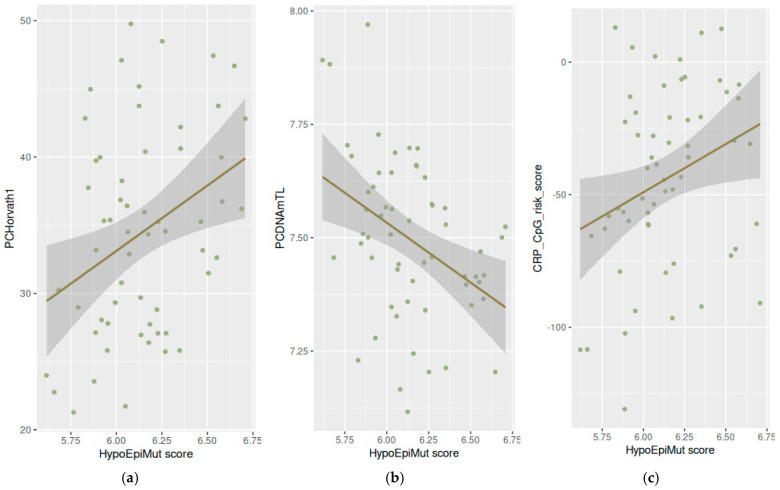
Correlation between the HypoEpiMut score and epigenetic aging surrogates: (**a**) PCHorvath1 (**b**), PCDNAmTL, and (**c**) CRP_CpG_risk_score. Each dot corresponds to a single sample, continuous lines represent linear regression trends and the shadows outline standard error boundaries.

**Figure 5:**
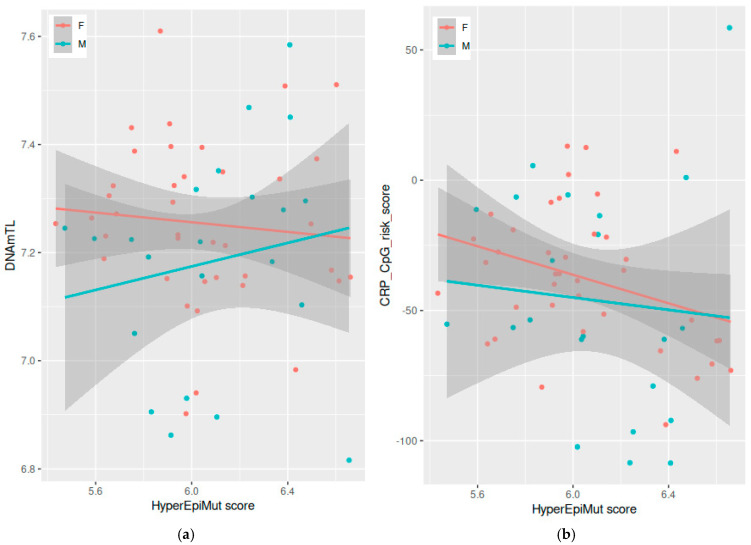
Correlation between the HyperEpiMut score and epigenetic aging surrogates: (**a**) DNAmTL and (**b**) CRP_CpG_risk_score in females and males. Each dot corresponds to a single sample, continuous lines represent linear regression trends for each sex group and the shadows outline respective standard error boundaries.

Analyzing females and males together, we found a significant correlation between the HypoEpiMut scores and the following three epigenetic clocks: PCHorvath1 (*p*-value = 0.001), PCDNAmTL (*p*-value = 0.005), and CRP_CpG_risk_score (*p*-value = 0.018) (Figure 4), and these findings were reproduced in the male population (PCHorvath1 *p*-value = 0.008, PCDNAmTL *p*-value = 0.017, and CRP_CpG_risk_score *p*-value = 0.038) (Supplementary Figure S12). Additionally, the HyperEpiMut score was correlated with the DNAmTL score in males (*p*-value = 0.027) (Figure 5a) and with the CRP_CpG_risk_score in females (*p*-value = 0.034) (Figure 5b).

## 4. Discussion

In a previous study, we investigated the possible association of the DNA methylation of isolated B cells with the responsiveness to HBV vaccination [3]. In this study, we deepened this topic by investigating, in the same cohort, the possible role of epigenetic drift, measured as the number of epivariants per individual in isolated B cells of individuals responding or non-responding to HBV vaccination. This method has the great advantage of providing an individual variance evaluation, thus allowing us to foresee possible clinical applications. We identified CpG sites with extreme aberrant methylation levels and considered their distribution, prevalence, and putative consequences on genes and pathways in order to study their potential role in the response to the vaccine. In fact, the results reported here provide evidence on the epigenetic differences between the R and NR groups on the global, chromosomal, and genetic levels.

Epigenetic drift is a phenomenon that increases exponentially with aging. Gentilini et al. [5] demonstrated that epigenetic changes, such as DNA methylation alterations, gradually accumulate over a lifetime, with an accelerated pace in older age. This process is influenced by various environmental and genetic factors, leading to stable alterations in epigenetic profiles. In examining the relationship between epigenetic drift and unresponsiveness to the HBV vaccine, we have appropriately adjusted the data for age. This adjustment is crucial, as it allows for isolating the effect of epigenetic drift independently from the simple passage of time. As a result, any association identified between epigenetic drift and vaccine response is corrected for age-related epigenetic changes, making the findings more reliable for specifically assessing the influence of epigenetic drift on immune response to the vaccine. Therefore, applied approach is significant, as it differentiates age-related epigenetic changes from those that might specifically impact vaccine responsiveness, potentially indicating that certain age-associated epigenetic modifications could interfere with immune response efficacy.

Recently, we reported a higher methylation variability in NRs compared to Rs [3]. The main finding that emerged from this further refinement of the analysis was the accumulation of epivariants in NRs with a significant increase in hyper-methylated epivariants. Contrary to a previous study in which we observed traits of epigenetic aging also in a sex-specific analysis, here, the differences in epivariant scores between female and male, and Rs and NRs, were not significant. However, the trend that the differences are more marked among males than in females is in line with what has already been previously presented [3], even though reaching the level of statistical significance is restricted by the sample size. Furthermore, it should be noted that variance analysis and epimutation analysis are two distinct methods to evaluate different aspects of the drift of the epigenetic architecture of cells.

We studied the distribution of the epivariations across the genome in order to assess whether it is possible to identify specific differences in the accrual of the epigenetic lesions. We did not find any difference in the distribution of the epivariants in the different functional areas of the genome. On the contrary, when we investigated the distribution of epigenetic drift in the chromosomes, we observed that chromosomes 1, 3, 11, 12, and 14 showed significant increases in epigenetic drift. In more detail, we observed that, in almost all of the chromosomes, there was a trend toward a higher number of hyper-methylated variants in NRs, with a significant increase in chromosomes 12 and 20. We further investigated this result and studied the distribution of epivariants in the genetic regions of these five chromosomes. We found 29 genes with an accrual of epivariants in NRs. Interestingly, many of these genes, such as *IRAK4*, *RUSC1_AS1*, *TROVE2*, or *MCCC1*, are involved in immunological functions [16,17,18,19]. We repeated the chromosome enrichment analysis in the two sexes separately and found the sex-specific chromosomal enrichment of such genetic drift. In detail, we found that female NRs were enriched in the total number of epivariants on chromosomes 10, 11, and 21, and male NRs in hyper-methylated epivariants on chromosomes 7, 12, 14, and 17. The gene analysis allowed us to identify two genes for females and five genes for males (Table 3), and some of them (such as *PROSER2_AS1* or *DCHS1*) are involved in the functions of the immune system [20,21], suggesting that the accumulation of epivariants has effects on the immune function of B cells.

To further corroborate this idea, the results of a pathway enrichment analysis confirm that genes enriched in epivariants that are not directly involved in the immune function are nevertheless involved in processes closely related to the correct functioning of the immune system. For example, altered glycan profiles were linked to many immune-related diseases, and glyco-based therapies to shape immune responses are emerging [22]. Steroid hormones were shown to have an important role in the functioning of the immune system that is bi-directional: the immune cells produce and respond to steroid hormones [23]. Peroxisomes were identified as key regulators of immune functions and inflammation [24]. The FoxO subfamily of transcription factors is responsible for the homeostasis and development of immune-relevant cells [25]. TNF signaling controls the typical immune response through the regulation of a range of pathways, including an immediate inflammatory reaction, with significant innate immune involvement among others [26]. Altogether, these results provide a possible mechanistic link between epigenetic drift and poor vaccination response.

In the previous study, we also found an association between responsiveness to HBV vaccination and epigenetic aging [3]. In the present analysis, we observed a positive correlation between epigenetic drift expressed with Shannon’s entropy and the HypoEpiMut score in the male population. Several epigenetic aging surrogates were correlated with epivariant scores. The increase in methylation age calculated with Horvath’s method was accompanied by an increase in the load of hypo-methylated aberrations. Interestingly, higher chronic low-grade inflammation, expressed by a model build on serum levels of C-reactive protein (CRP), was accompanied by higher hypo-methylated and lower hyper-methylated epivariant loads. A similar tendency was observed for surrogates of the age-related telomere length: an increased shortening linked to higher HypoEpiMut scores and lower HyperEpiMut scores. All of these results show that, as expected, these epigenetic dynamics are indeed interconnected, although these interconnections seem not to be involved in the altered immunological activity, since they present indifferently in Rs and NRs.

The principal value of this study lies in the unique biological material that has been assessed—isolated B cells. It allowed us to work on a data of a high quality, with a minimized impact of noise. However, it must be noted that this approach requires much more work and time, and is cost-consuming compared common whole blood/PBMC assessments. Therefore, this framework might be achieved but at the expense of the number of recruited participants and collected samples. Another weakness of this experimental design is the fact that B cells are not an abundant subpopulation in the peripheral blood, which constrains the generation of data on multiple molecular levels and impedes multi-omics approaches to integrate the data. Thus, the following are the future research directions: to increase the numerosity of the collected samples and to extend the analysis with additional assays in order to link the described epivariations to functional aspects, which, hopefully, would provide mechanistic insights leading to improvements in the design of vaccination strategies and protocols.

Overall, these findings provide additional evidence on the possible contribution of DNA methylation mechanisms to immune response after vaccination against HBV. Epigenetic drift is one of the molecular dynamics that is involved in aging, since it has been demonstrated that the number of epivariations increases exponentially with aging [5]. The significant increase in epigenetic drift in B cells of NRs further supports the idea that such B cells are epigenetically older than those of responder individuals, and suggests that an age-related derangement of the epigenetic architecture is involved in the poor HBV vaccination response. As a final consideration, in general, somatic genetic variability is presumed to have a greater weight in epivariants. Therefore, the B cells of NRs are genetically more unstable, which is a trait accompanying natural aging, and which, in turn, is in line with a loss of functional efficiency.

## Figures and Tables

**Table 1:** Demographics of studied cohort.

Characteristics	NRs (*n* = 30)	Rs (*n* = 41)	*p*-Value
Sex, F	15 (50%)	27 (66%)	0.272 ^3^
Age ^1^	35.93 (11.70)	33.45 (7.86)	0.319 ^4^
B cell count ^2^	2.75 × 10^6^ (2.12 × 10^6^)	2.10 × 10^6^ (2.10 × 10^6^)	0.149 ^5^

^1^ Mean (standard deviation); ^2^ Median (IQR); ^3^ X^2^ test; ^4^ *t*-test; ^5^ Wilcoxon test.

**Table 2:** Genes differentially enriched in epivariant scores between Rs and NRs.

Gene	Chr	Epivariants	Mean R	Mean NR	*p*-Value ^1^	Adj. *p*-Value ^2^
*WDR66*	12	hypo + hyper	0.200	0.458	0.022	0.995
*ING4*	12	hypo + hyper	0.086	0.333	0.033	0.995
*PUS7L*	12	hypo + hyper	0.343	1.042	0.043	0.995
*IRAK4*	12	hypo + hyper	0.343	1.042	0.043	0.995
*THTPA*	14	hypo + hyper	0.086	0.250	0.010	0.997
*ZFHX2*	14	hypo + hyper	0.086	0.208	0.018	0.997
*SIPA1L1*	14	hypo + hyper	0.086	0.250	0.035	0.997
*RUSC1-AS1*	1	hypo + hyper	0.086	0.292	0.009	0.995
*UCHL5*	1	hypo + hyper	0.029	0.208	0.020	0.995
*EMC1*	1	hypo + hyper	0.029	0.167	0.027	0.995
*MRTO4*	1	hypo + hyper	0.029	0.167	0.027	0.995
*TROVE2*	1	hypo + hyper	0.029	0.167	0.030	0.995
*CYP2J2*	1	hypo + hyper	0.029	0.417	0.035	0.995
*EPHB2*	1	hypo + hyper	0.029	0.208	0.041	0.995
*DENND2C*	1	hypo + hyper	0.057	0.167	0.048	0.995
*BDNF-AS*	11	hypo + hyper	0.029	0.208	0.014	0.996
*LIN7C*	11	hypo + hyper	0.029	0.208	0.014	0.996
*TBX10*	11	hypo + hyper	0.086	0.292	0.044	0.996
*CORO1B*	11	hypo + hyper	0.029	0.083	0.050	0.996
*SOX2-OT*	3	hypo + hyper	0.086	0.375	0.007	0.998
*MME*	3	hypo + hyper	0.029	0.167	0.018	0.998
*MCCC1*	3	hypo + hyper	0.771	3.000	0.025	0.998
*AHSG*	3	hypo + hyper	0.029	0.167	0.038	0.998
*CEP63*	3	hypo + hyper	0.029	0.208	0.038	0.998
*PLSCR1*	3	hypo + hyper	0.257	0.667	0.041	0.998
*KCNAB1*	3	hypo + hyper	0.029	0.250	0.050	0.998
*CKAP4*	12	hyper	0.056	0.200	0.012	0.994
*WDR66*	12	hyper	0.139	0.400	0.042	0.994
*B4GALT5*	20	hyper	0.028	0.200	0.027	0.996

^1^ Z-test; ^2^ Multiple test correction with the Benjamini–Hochberg approach.

**Table 3:** Sex-specific genes differentially enriched in epivariant scores between Rs and NRs.

Gene	Chr	Epivariants	Sex	Median R	Median NR	*p*-Value ^1^	Adj. *p*-Value ^2^
*PROSER2-AS1*	10	hypo + hyper	F	0.074	0.400	0.012	0.997
*DCHS1*	11	hypo + hyper	F	0.037	0.133	0.020	0.999
*ZNF232*	17	hyper	M	0.500	0.867	0.047	1.000
*SLC37A3*	7	hyper	M	0.214	0.600	0.045	1.000
*PPP1R13B*	14	hyper	M	0.071	0.467	0.041	1.000
*LINC00637*	14	hyper	M	0.071	0.467	0.041	1.000
*RTN1*	14	hyper	M	0.071	0.600	0.045	1.000

^1^ Z-test; ^2^ Multiple test correction with the Benjamini–Hochberg approach.

**Table 4:** KEGG pathways significantly enriched (*p*-value < 0.05) in NR-exclusive epivariant genes.

Pathway	Overlap ^1^	*p*-Value ^2^	Combined Score ^3^
Other glycan degradation	7/18	0.009	17.656
Pentose and glucuronate interconversions	10/34	0.018	9.916
Ascorbate and aldarate metabolism	9/30	0.022	9.767
Steroid hormone biosynthesis	14/61	0.049	5.355

^1^ Ratio between the number of genes provided as the input list and present in a pathway and the total number of genes constituting that pathway. ^2^ Fisher’s exact test. ^3^ Score computed by taking the logarithm of the *p*-value from Fisher’s exact test and multiplying that by the z-score of the deviation from the expected rank. Rank-based ranking is derived from running Fisher’s exact test for many random gene sets in order to compute a mean rank and standard deviation from the expected rank for each term in the gene set library, and finally calculating a z-score to assess the deviation from the expected rank.

**Table 5:** KEGG pathways significantly enriched (*p*-value < 0.05) in R-exclusive epivariant genes.

Pathway	Overlap ^1^	*p*-Value ^2^	Combined Score ^3^
Peroxisome	30/82	0.006	9.398
Small cell lung cancer	31/92	0.020	6.385
Pancreatic cancer	26/76	0.026	6.103
TNF signaling pathway	36/112	0.027	5.486
FoxO signaling pathway	41/131	0.030	5.132
Cell cycle	39/124	0.031	5.110
Mitophagy	23/68	0.039	5.309
Colorectal cancer	28/86	0.040	4.976
Chronic myeloid leukemia	25/76	0.045	4.875
Salmonella infection	71/249	0.048	3.896

^1^ Ratio between the number of genes provided as the input list and present in a pathway and the total number of genes constituting that pathway. ^2^ Fisher’s exact test. **^3^** Score computed by taking the logarithm of the *p*-value from Fisher’s exact test and multiplying that by the z-score of the deviation from the expected rank. Rank-based ranking is derived from running Fisher’s exact test for many random gene sets in order to compute a mean rank and standard deviation from the expected rank for each term in the gene set library, and finally calculating a z-score to assess the deviation from the expected rank.

## Data Availability

The data that support the findings of this study are available from the corresponding authors, K.K. and P.G., upon reasonable request.

## Supplementary Materials

The following supporting information can be downloaded at: https://www.mdpi.com/article/10.3390/vaccines12121330/s1, Figure S1: Age distribution in the studied cohort (a) in the overall population, (b) by phenotype, and (c) by the sex group; Figure S2: Number of (a) hypo- and (b) hyper-epivariants in the R and NR groups per sample; Figure S3. Distribution of epivariants in Rs and NRs in relation to (a) the CpG island and (b) gene; Figure S4: Distribution of hypo- and hyper-epivariants in Rs and NRs in relation to (a) the CpG island and (b) gene; Figure S5. Distribution of (a) hypo- and (b) hyper-epivariants in Rs and NRs in relation to the CpG island; Figure S6. Distribution of (a) hypo- and (b) hyper-epivariants in Rs and NRs in relation to the gene; Figure S7. Boxplot of (a) the TotEpiMut score and (b) the HypoEpiMut score in Rs and NRs; Figure S8. Boxplot of (a) the TotEpiMut, (b) HypoEpiMut, and (c) HyperEpiMut scores in Rs and NRs in the female population; Figure S9. Boxplot of (a) the TotEpiMut, (b) HypoEpiMut, and (c) HyperEpiMut scores in Rs and NRs in the male population; Figure S10. Differences in the chromosomal level between Rs and NRs in (a) the TotEpiMut and (b) HyperEpiMut scores; Figure S11. Boxplot of Shannon’s entropy in (a) the entire cohort, (b) the female population, and (c) the male population; Figure S12. Correlation between the HypoEpiMut score and epigenetic aging surrogates for the (a) PCHorvath1, (b) PCDNAmTL, and (c) CRP_CpG_risk_score in the male population; Table S1: Characterization and differences between epigenetic drift, epigenetic shift, antigenic drift, and antigenic shift; Table S2: Distribution of epivariants in Rs and NRs in relation to the gene and CpG island; Table S3: Results of the differential analysis on the chromosomal level; Table S4: Results of the differential analysis on the chromosomal level in the female population; Table S5: Results of the differential analysis on the chromosomal level in the male population; Table S6: Results of the correlation analysis.
