# Supplementary material for: Epigenetic Drift Is Involved in the Efficacy of HBV Vaccination
Source: Vaccines (Basel). 2024 Nov 27;12(12):1330. doi: 10.3390/vaccines12121330 (PMC11680278; doi:10.3390/vaccines12121330)
Supplement: Supplementary file 1 [file vaccines-12-01330-s001.zip › SupplementaryFigures.pdf]

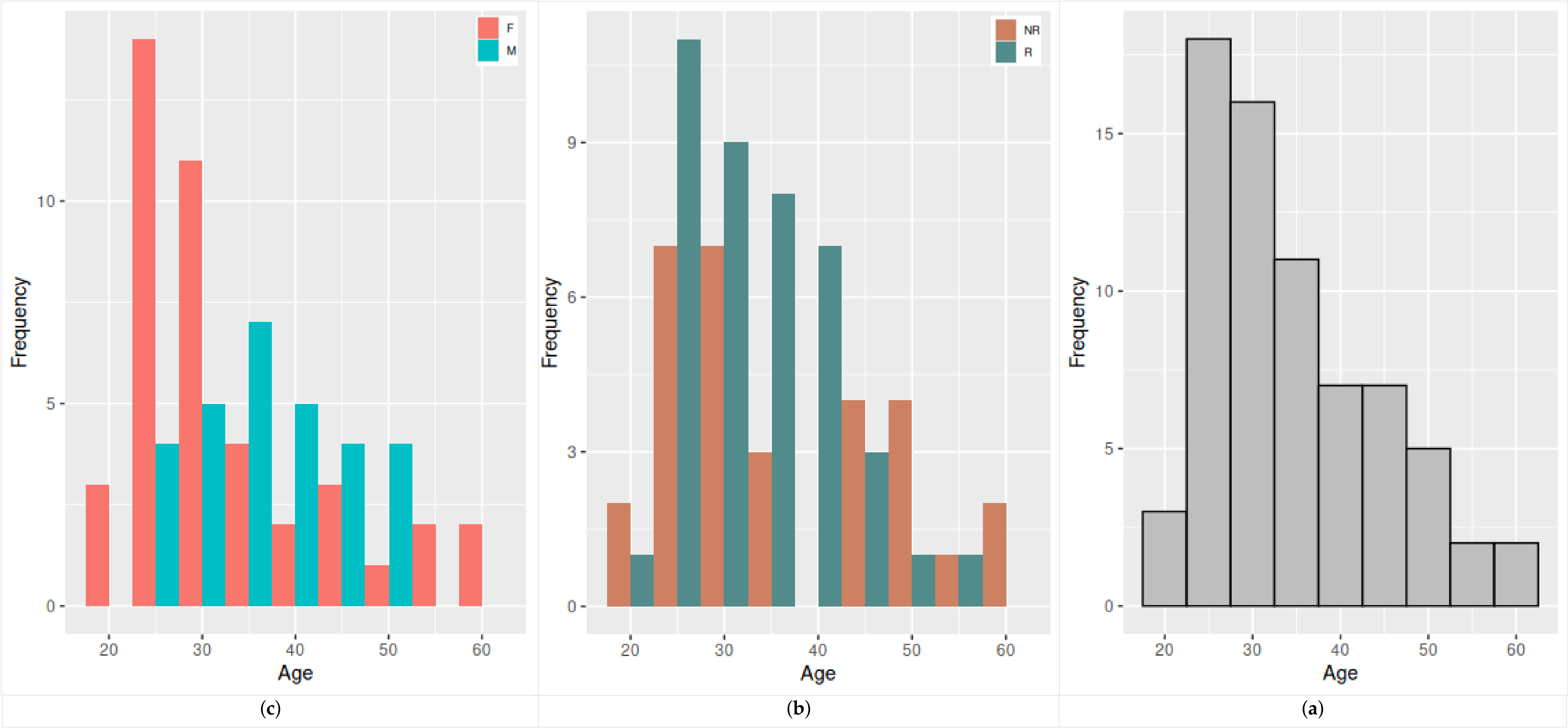


**Figure S1.** Age distribution in studied cohort: (**a**) in overall population; (**b**) by phenotype and (**c**) by sex group.

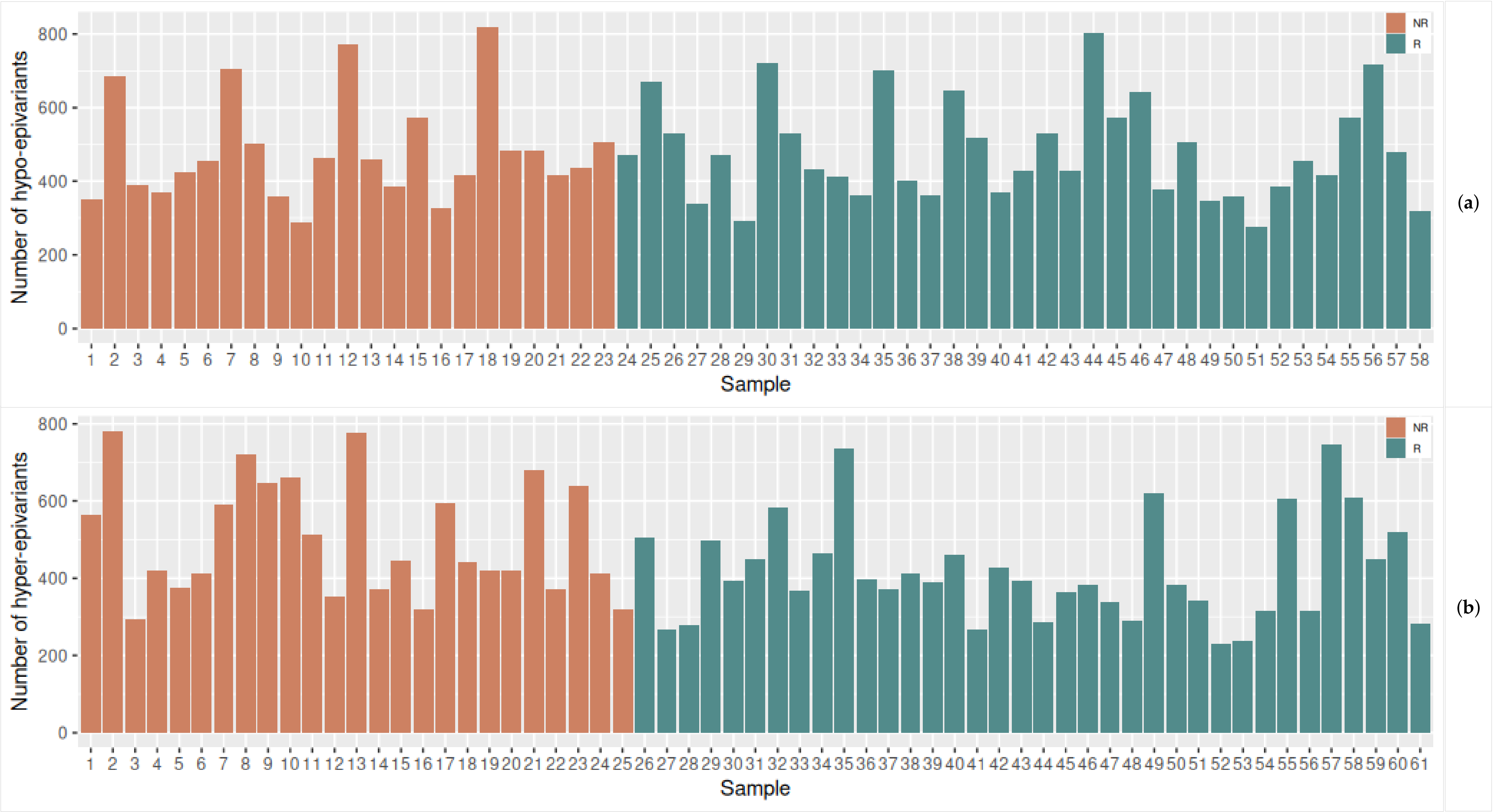


**Figure S2.** Number of: (**a**) hypo- and (**b**) hyper-epivariants in R and NR group per sample.

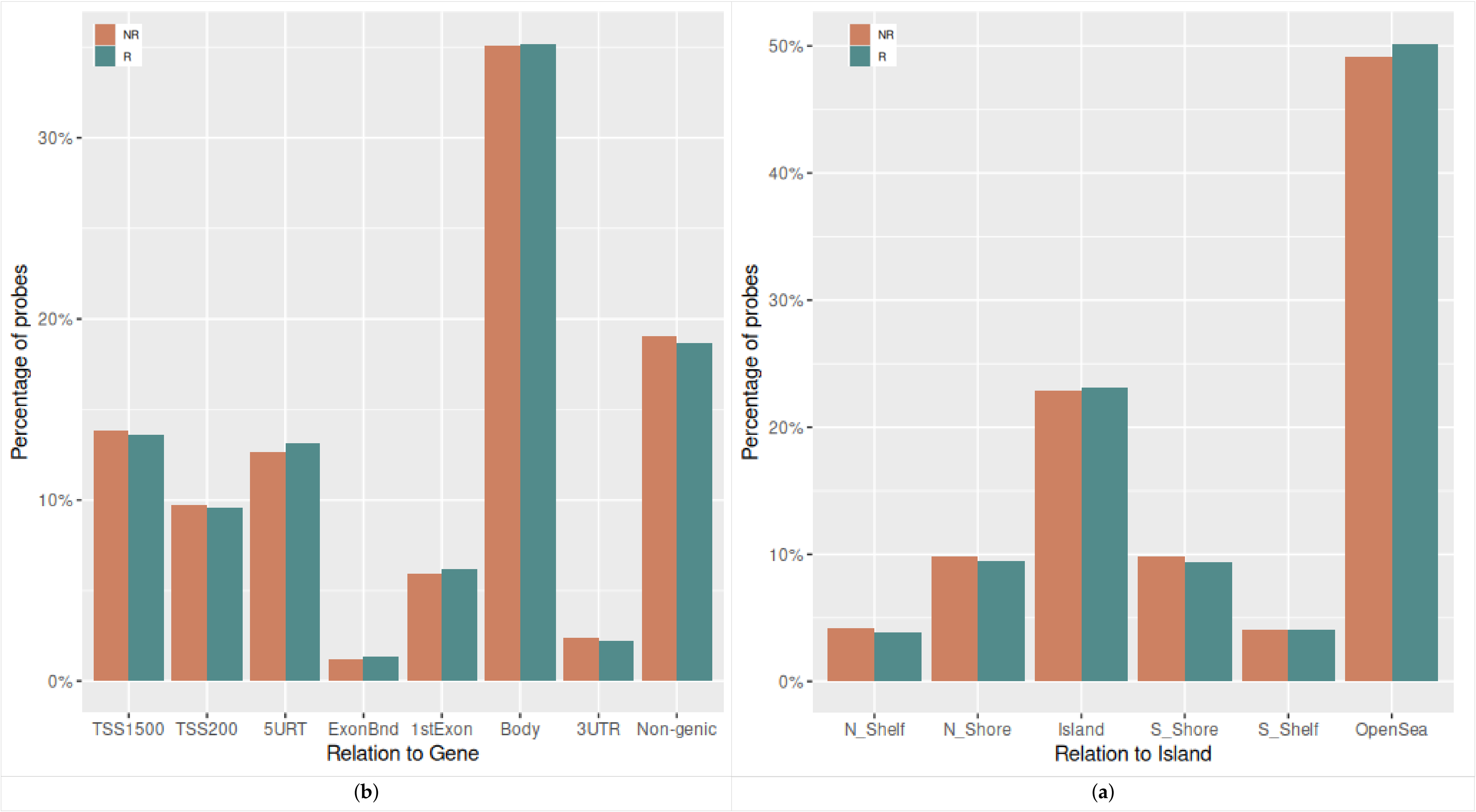


**Figure S3.** Distribution of epivariants in R and NR in relation to: (**a**) CpG island and (**b**) gene.

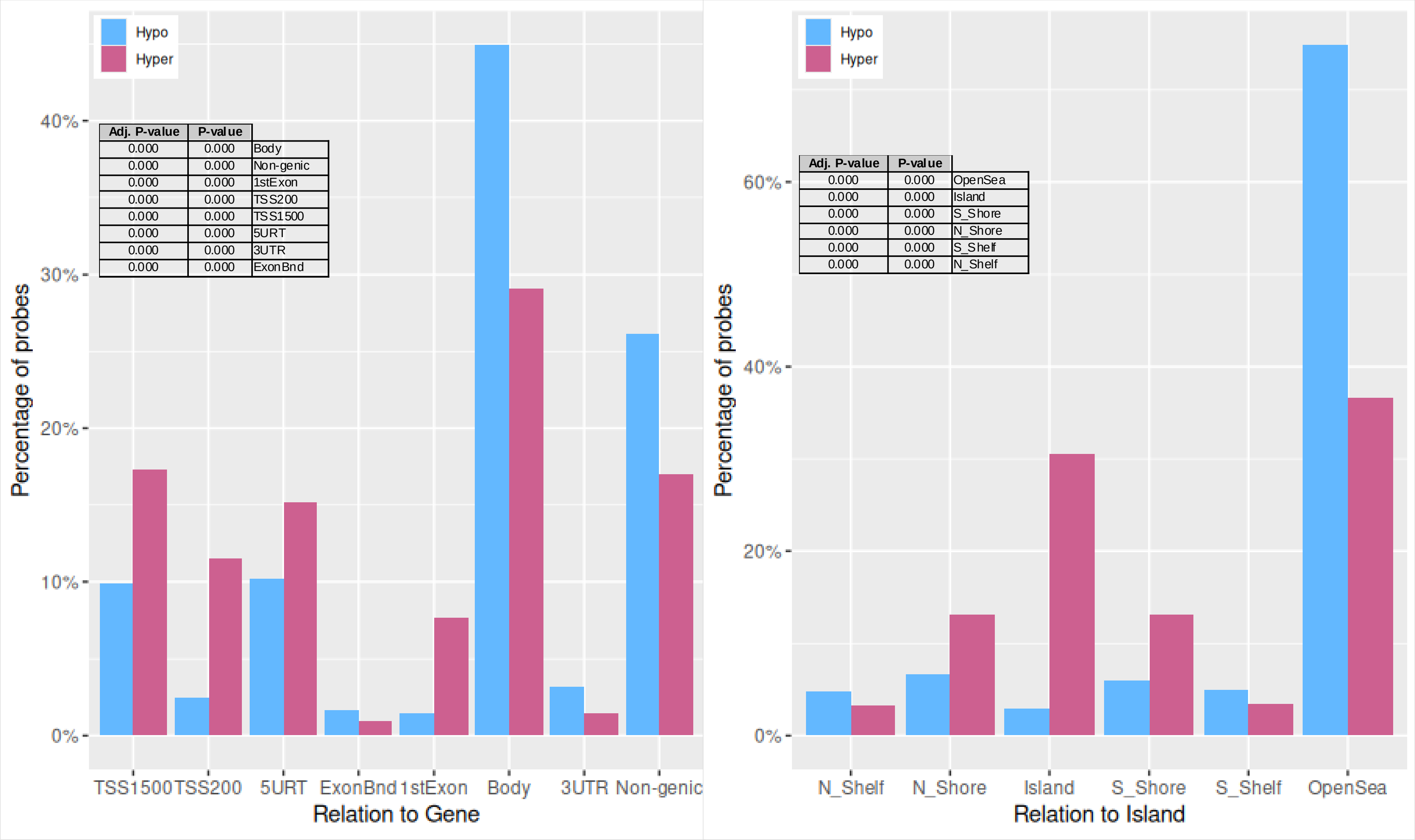


(**b**) (**a**)

**Figure S4.** Distribution of hypo- and hyper-epivariants in R and NR in relation to: (**a**) CpG island and (**b**) gene. In tables p-values from $X^2$ test are reported.

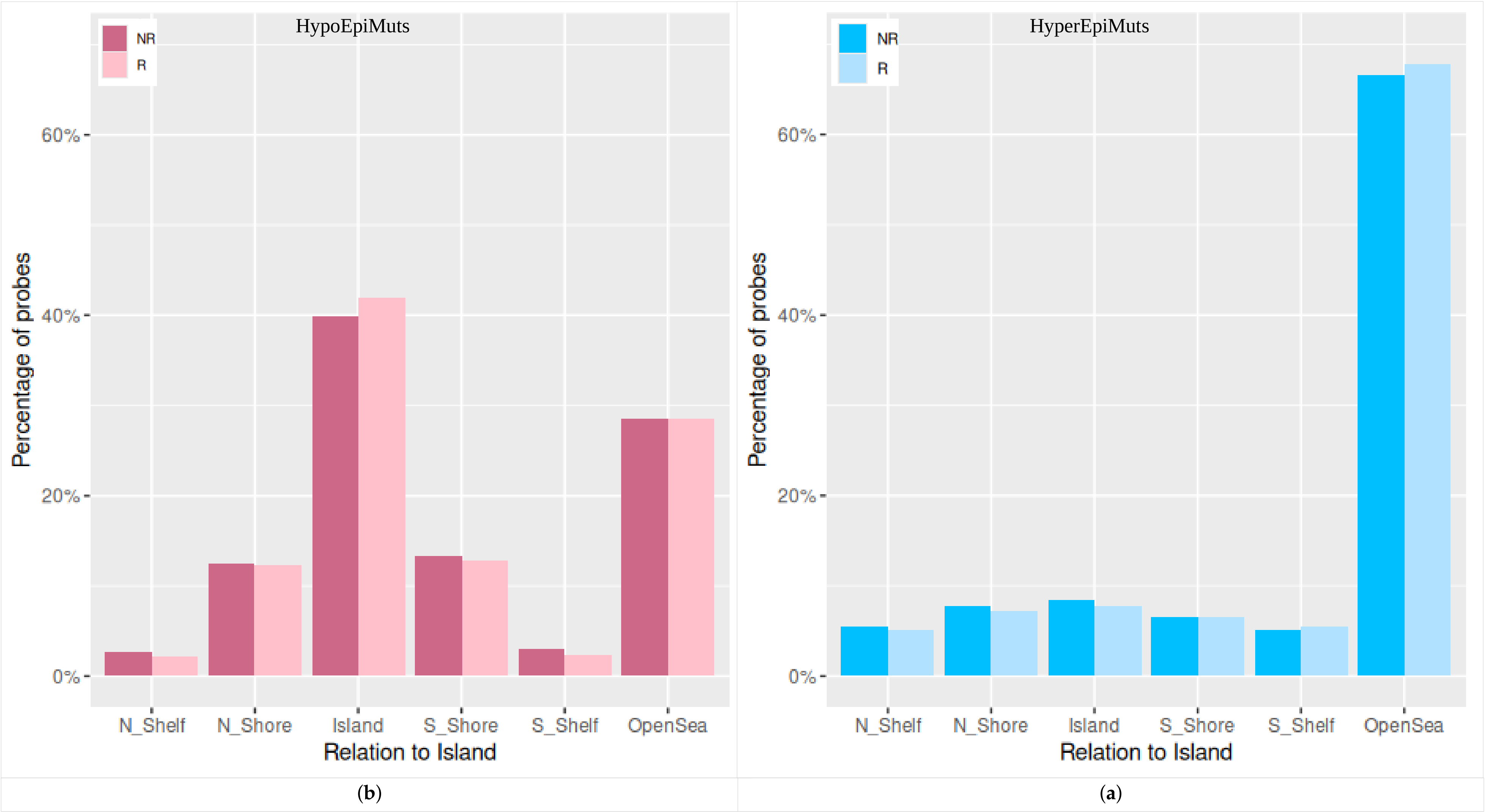


**Figure S5.** Distribution of: (**a**) hypo- and (**b**) hyper epivariants in R and NR in relation to CpG island.

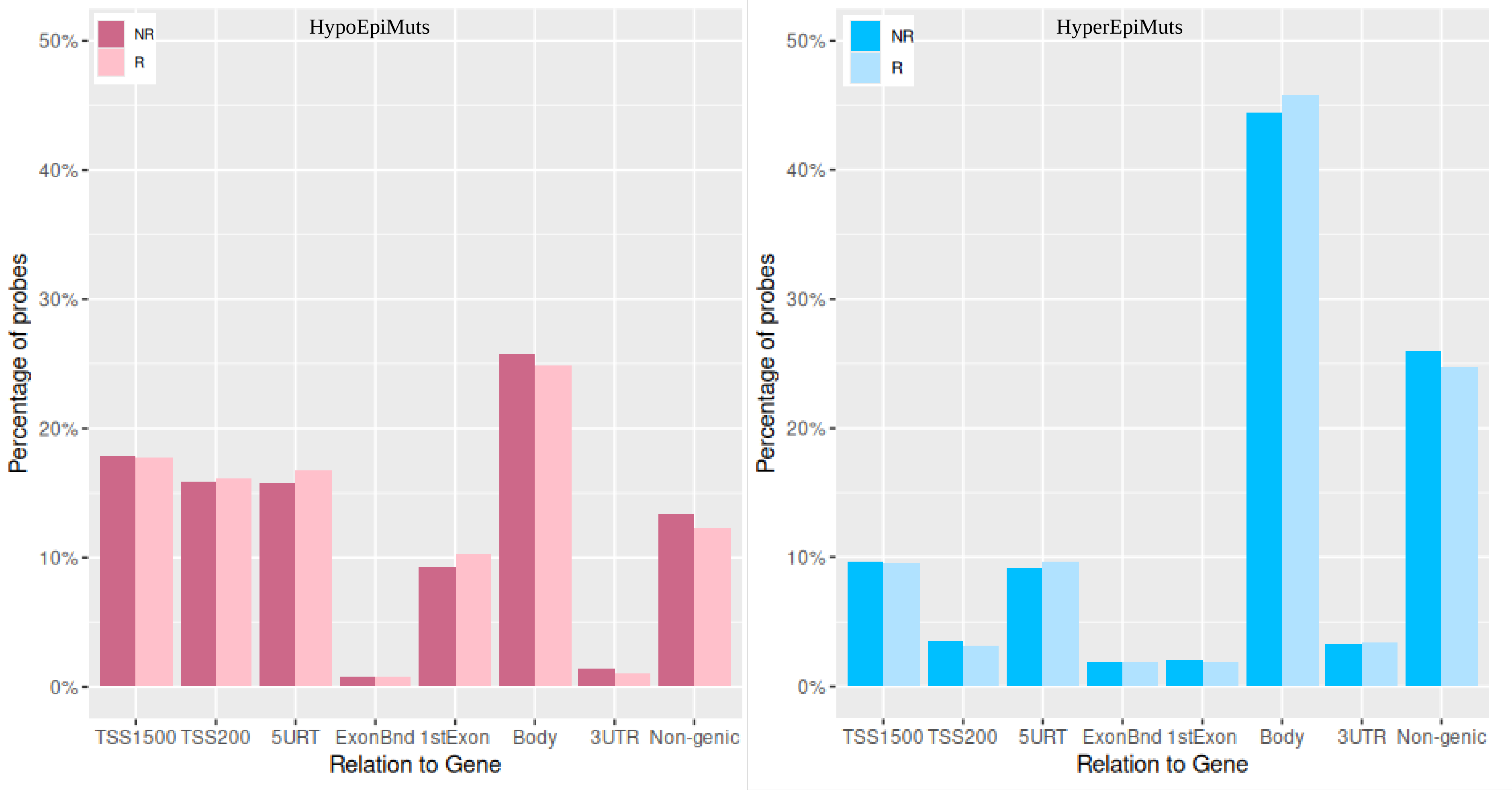


(**b**) (**a**)

**Figure S6.** Distribution of: (**a**) hypo- and (**b**) hyper epivariants in R and NR in relation to gene.

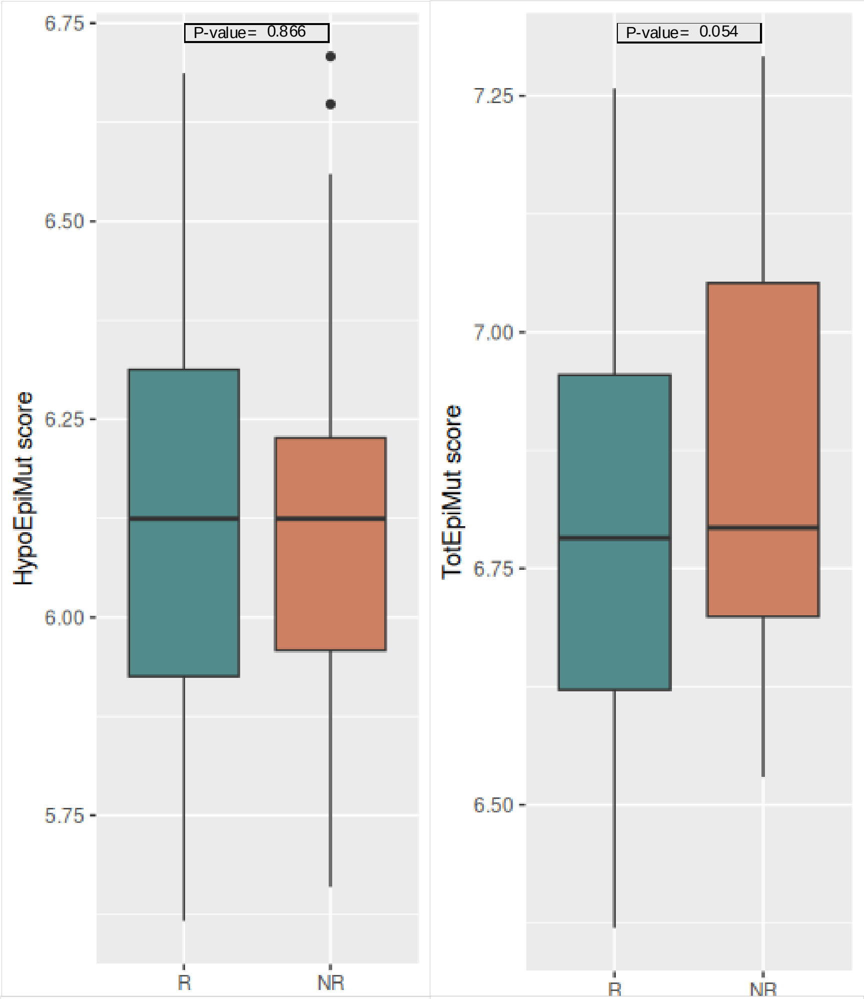


(b) (a)

**Figure S7.** Boxplot of: (**a**) TotEpiMut score and (**b**) HypoEpiMut score in R and NR. P-values from glm model are reported.

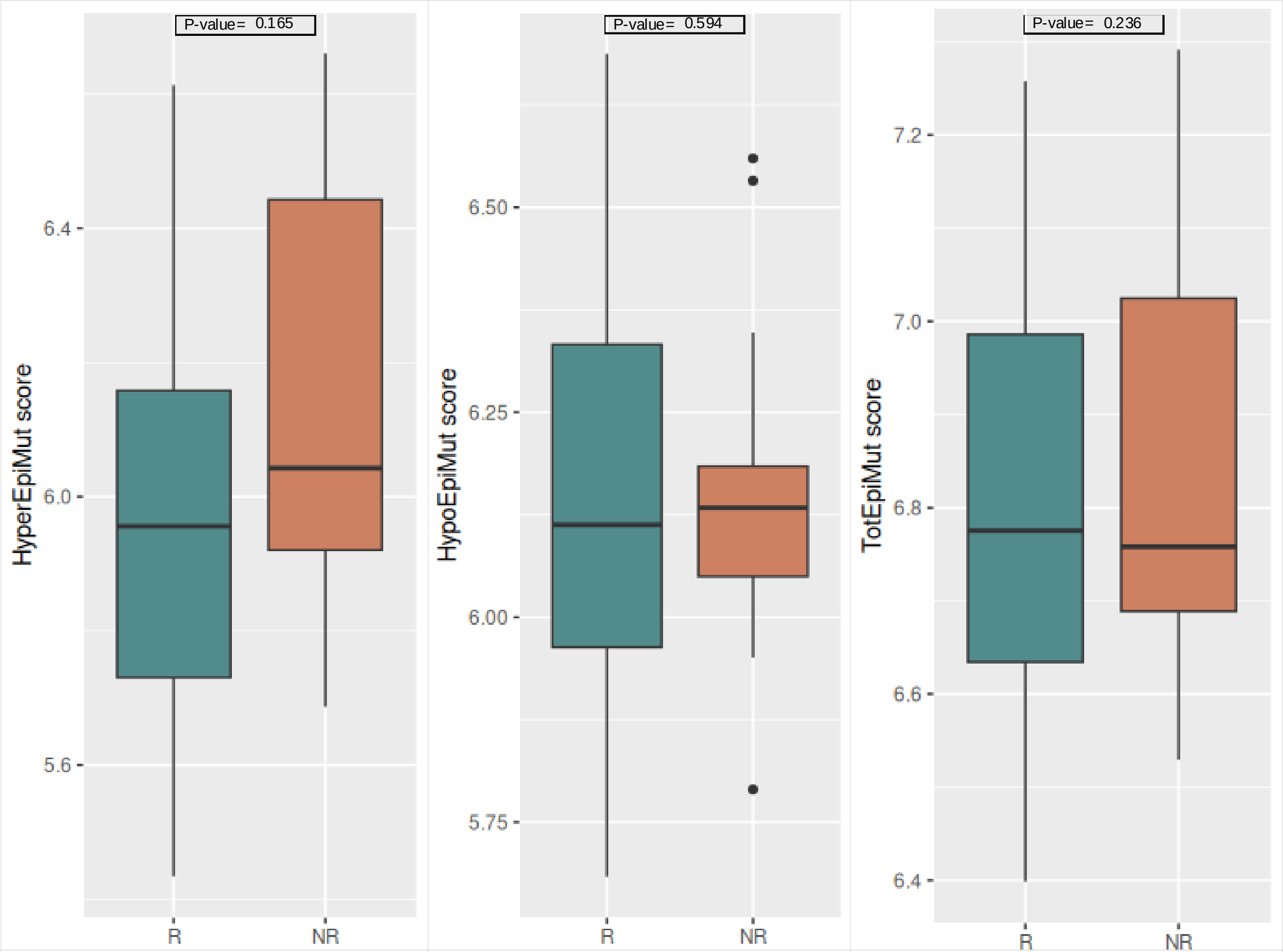


(c) (b) (a)

**Figure S8.** Boxplot of: (**a**) TotEpiMut, (**b**) HypoEpiMut and (**c**) HyperEpiMut scores in R and NR in female population. P-values from glm model are reported.

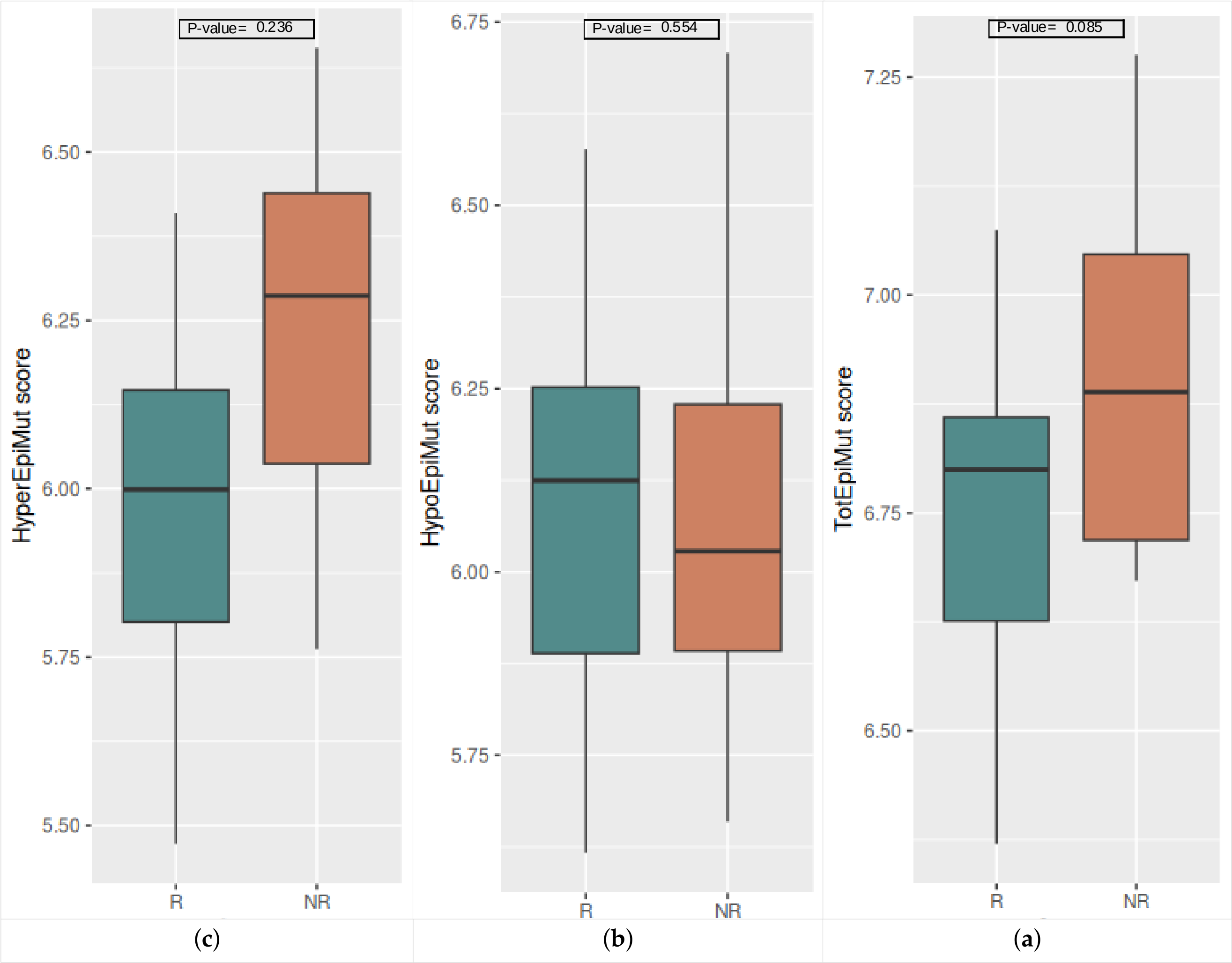


**Figure S9.** Boxplot of: (**a**) TotEpiMut, (**b**) HypoEpiMut and (**c**) HyperEpiMut scores in R and NR in male population. P-values from glm model are reported.

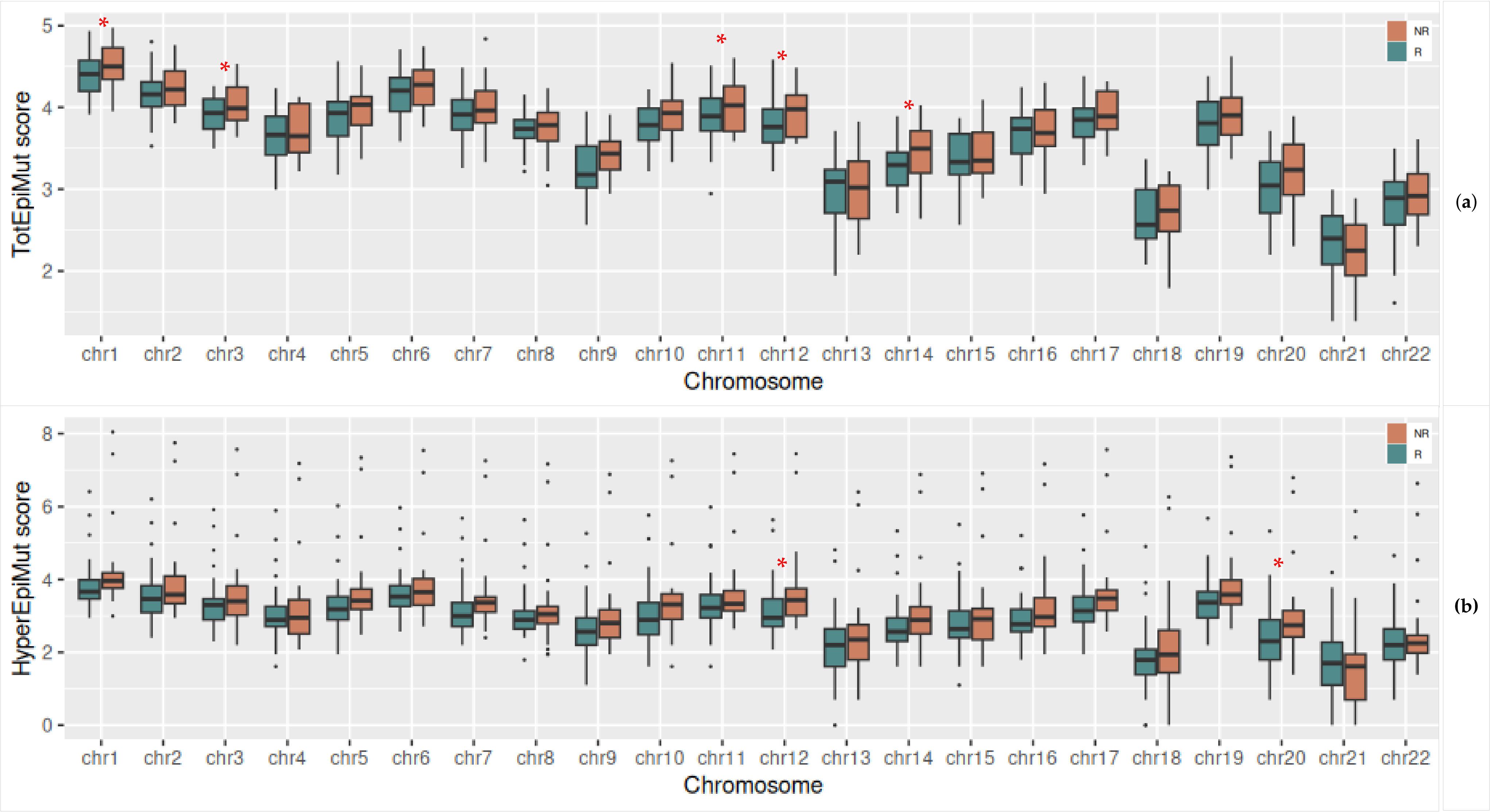


**Figure S10.** Differences on chromosomal level between R and NR in: (**a**) TotEpiMut and (**b**) HyperEpiMut scores. Asterisk marks significant differences.

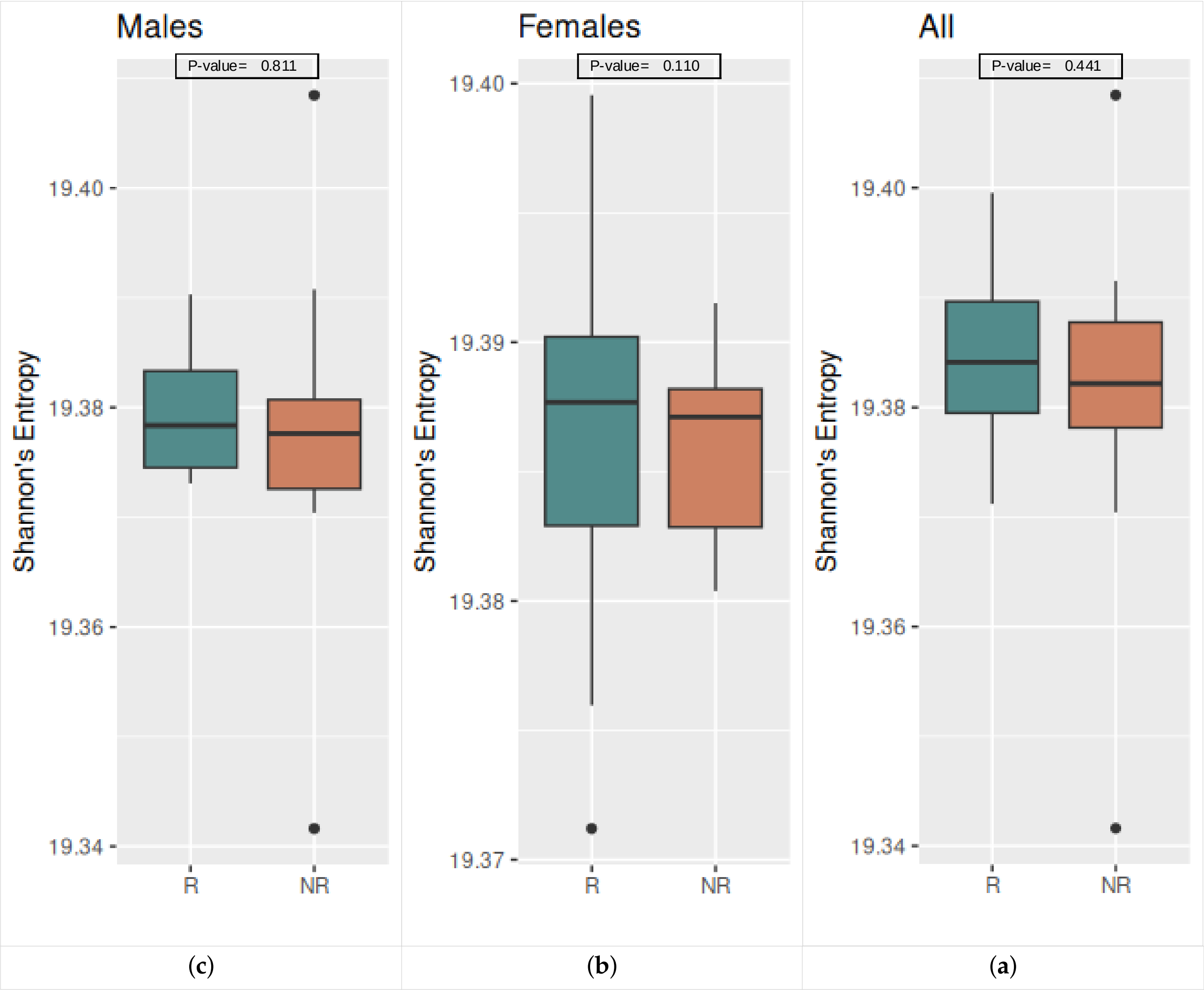


**Figure S11.** Boxplot of Shannon's entropy in: (**a**) entire cohort, (**b**) females population and (**c**) male population. P-values from glm model are reported.

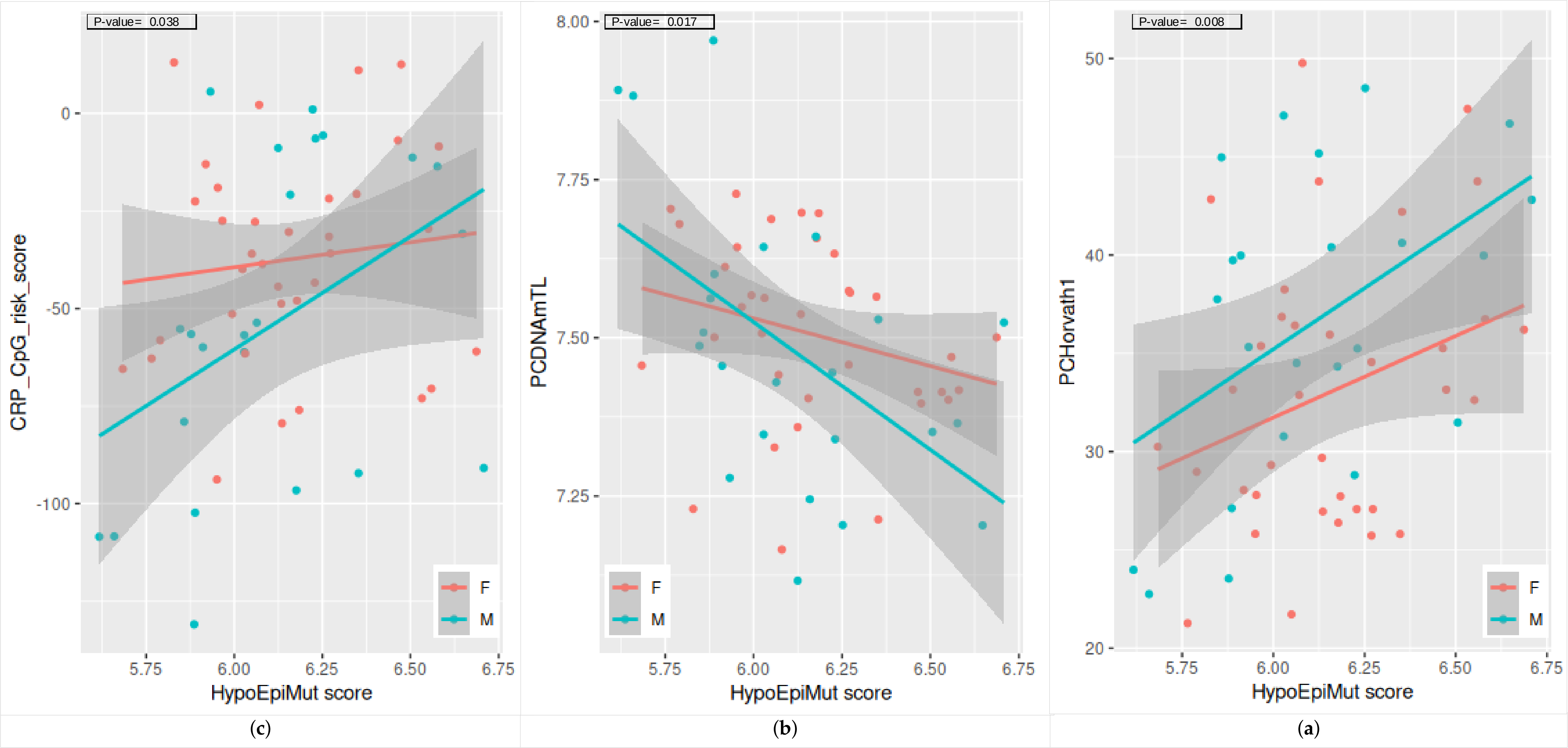


**Figure S12.** Correlation between HypoEpiMut score and epigenetic aging surrogates: (a) PCHorvath1 (b) PCDNAmTL and (c) CRP_CpG_risk_score in male population. P-values from glm model are reported.
